# Universal Critical Behaviours in Non-Hermitian Phase Transitions

**DOI:** 10.1038/s41598-017-07344-z

**Published:** 2017-08-02

**Authors:** Bo-Bo Wei, Liang Jin

**Affiliations:** 1 School of Physics and Energy, Shenzhen University, Shenzhen, 518060 China; 2 School of Physics, Nankai University, Tianjin, 300071 China

## Abstract

Quantum phase transitions occur in non-Hermitian systems. In this work we show that density functional theory, for the first time, uncovers universal critical behaviors for quantum phase transitions and quantum entanglement in non-Hermitian many-body systems. To be specific, we first prove that the non-degenerate steady state of a non-Hermitian quantum many body system is a universal function of the first derivative of the steady state energy with respect to the control parameter. This finding has far-reaching consequences for non-Hermitian systems. First, it bridges the non-analytic behavior of physical observable and no-analytic behavior of steady state energy, which explains why the quantum phase transitions in non-Hermitian systems occur for finite systems. Second, it predicts universal scaling behaviors of any physical observable at non-Hermitian phase transition point with scaling exponent being (1 − 1/*p*) with *p* being the number of coalesced states at the exceptional point. Third, it reveals that quantum entanglement in non-Hermitian phase transition point presents universal scaling behaviors with critical exponents being (1 − 1/*p*). These results uncover universal critical behaviors in non-Hermitian phase transitions and provide profound connections between entanglement and phase transition in non-Hermitian quantum many-body physics.

## Introduction

Quantum phase transitions occurs when the ground state of a quantum many-body system experiences a sudden change as the parameter of the system is tuned through a critical point^1^. It is one of the most intriguing phenomena in many-body physics because it indicates emergence of new states of quantum matter and new physics^1, 2^. In the study of quantum phase transitions, it is usually assumed that the Hamiltonians are Hermitian. However the non-Hermitian Hamiltonian indeed arises due to the spontaneous decay in current experimental results in cavities^3, 4^, waveguides^5, 6^, optomechanics^7^ and cold atoms^8^. These experimental progresses provide new opportunity for discovering new classes of phase transitions beyond the Hermitian paradigm.

Non-Hermitian models draw a great deal of interest since they present richer behaviors^9–15^, particularly in the PT symmetry optical systems^16–18^. The intriguing phenomena include PT symmetry breaking and power oscillation^19, 20^, coherent perfect absorption^21, 22^, unidirectional reflectionless and invisibility^23^, the gain induced large optical nonlinear^24^, and the single-mode PT symmetric lasing^25^. In PT symmetric Su-Schrieffer-Heeger chain, both topological protected PT symmetric interface state^26^ and PT phase transition induced interface state can be used to realize robust light transport^27^. Dynamical phase transitions were demonstrated when the parameters are extended into the complex plane of physical parameters^28–31^. Recently, It was found that quantum phase transitions occurs in the steady state of non-Hermitian systems in specific models^32, 33^. However the universal critical behaviors of quantum phase transitions and of quantum entanglement in the steady state of a more general non-Hermitian system have been elusive.

The steady state for non-Hermitian Hamiltonians plays the same role as the ground state for Hermitian Hamiltonians. In this article we uncover the universal critical behavior of quantum phase transitions and of quantum entanglement in the steady state of non-Hermitian many-body systems from density functional theory. We rigorously prove that the non-degenerate steady state of a non-Hermitian quantum man-body system is a universal function of the first derivative of the steady state energy with respect to the control parameter. Furthermore, we show that quantum entanglement in the non-degenerate steady state is also a universal function of first derivative of the steady state energy with respect to the control parameter. Because the non-Hermitian phase transition points are the exceptional point of the Hamiltonian^9–14^, the first derivative of the steady state energy presents universal scaling behavior near the exceptional point^9, 34^. Due to the universal dependence of the steady state on the first derivative of the steady state energy, we deduce universal critical behaviors of physical observables and of quantum entanglement at non-Hermitian phase transitions point of the steady state.

## Results

### Quantum Phase Transitions in Non-Hermitian Systems

Let us consider a type of non-Hermitian quantum many-body system with Hamiltonian,

1 $$H(\lambda )={H}_{0}+i\gamma {H}_{1},$$

where *γ* is a real control parameter and *H*_0_ and *H*_1_ are Hermitian operators and we consider the most interesting case where [*H*_0_, *H*_1_] ≠ 0. To realize the non-Hermitian term, we assume that the one of the atomic state of a three level atom has a finite lifetime with linewidth *γ*. In the absence of a spontaneous decay event, the atoms evolution are governed by the Hamiltonian in Equation (1)^35–39^. In reality, one would perform the experiment many times and the experimental runs without decay event realize Equation (1)^32, 33^.

Non-Hermitian Hamiltonian described in Equation (1) has eigenstates with complex eigenvalues. An arbitrary state vector can be written as a superposition of the eigenstates of *H*. With time evolution, the state vector evolves under $\exp (-itH)$. Due to the non-Hermiticity of *H*, the weight in each eigenstate decreases over time because of the imaginary parts of the eigenvalues. After a sufficient amount of time, the state consists mostly of the eigenstate whose eigenvalue has the largest imaginary part. This eigenstate is termed the steady state and denoted by $|{{\rm{\Psi }}}_{S}\rangle$ and it satisfies the Schrödinger equation,

2 $$H(\gamma )|{{\rm{\Psi }}}_{S}(\gamma )\rangle ={E}_{S}(\gamma )|{{\rm{\Psi }}}_{S}(\gamma )\rangle .$$

We are interested in this surviving eigenstate, because it is the one that would be observed experimentally. Because *H*(*γ*) is non-Hermitian, the left eigen state of *H*(*γ*) satisfies that

3 $$\langle{\mathop{{\rm{\Psi }}}\limits^{ \sim }}_{S}(\gamma )|H(\gamma )={E}_{S}(\gamma {)}^{\ast }\langle{\mathop{{\rm{\Psi }}}\limits^{ \sim }}_{S}(\gamma )|.$$

For non-Hermitian Hamiltonian, it is more convenient to adopt the biorthogonal basis^9^. We normalize the left and right eigenvectors of the Hamiltonian by $\langle {\tilde{{\rm{\Psi }}}}_{S}(\gamma )|{{\rm{\Psi }}}_{S}(\gamma )\rangle =1$. Based on these concepts for non-Hermitian systems, we are ready to establish the first central theorem of this work.

### Theorem 1

The non-degenerate steady state of a non-Hermitian quantum many-body system with Hamiltonian *H*(*λ*) = *H*_0_ + *iγH*_1_ is a universal function of the first derivative of the steady state energy with respect to the control parameter, $\,\frac{\partial {E}_{S}}{\partial \gamma }$.

In Theorem 1, the universal means that the function form of the dependence of steady state on the first derivative of the energy does not change with variation of the control parameter as long as the steady state is in the same phase or does not experience any non-analytic point. The proof of Theorem 1 is given in the Methods. Theorem 1 is quite general and valid for any finite interacting spin systems, Fermions or Bosons in lattices. Theorem 1 is in the same spirit as density functional theory developed by Honhenberg, Kohn and Sham^40, 41^. Here we prove that the one-to-one correspondence between the steady state and the density is also valid in non-Hermitian systems for the first time.

An immediate consequence of the Theorem 1 is that the steady state average value of any physical observable *O* which does not commute with the Hamiltonian [*O*, *H*] ≠ 0 is also a universal function of the first derivative of the steady state energy with respect to the control parameter, $\,\frac{\partial {E}_{S}}{\partial \gamma }$ as

4 $$\langle O\rangle =\langle {{\rm{\Psi }}}_{S}(\tfrac{\partial {E}_{S}}{\partial \gamma })|O|{{\rm{\Psi }}}_{S}(\tfrac{\partial {E}_{S}}{\partial \gamma })\rangle .$$

This functional form is universal with respect to the control parameter as long as the steady state is in the same phase and non-degenerate.

Non-Hermitian phase transition point, also called exceptional point, where two or more energy levels coalesce^9^. We assume that *p* ≥ 2 levels coalesce at the exceptional point of a non-Hermitian system. Around the exceptional point, which is also an algebraic branch point, we can expand the steady state energy by

5 $${E}_{S}(\gamma )=\sum _{i=0}^{\infty }{\alpha }_{i}{(\gamma -{\gamma }_{c})}^{i/p}.$$

Here *α*_*i*_, *i* = 0, 1, 2, … are expansion coefficients. If *α*_1_ ≠ 0, we have

6 $$\frac{\partial {E}_{S}(\gamma )}{\partial \gamma }{|}_{\gamma \to {\gamma }_{c}}\propto {(\gamma -{\gamma }_{c})}^{(1-p)/p}.$$

It diverges as *γ* → *γ*_*c*_. Since the average value of any physical observable is a universal function of the first derivative of the steady state energy, defining *Y* ≡ $\,\frac{\partial {E}_{S}}{\partial \gamma }$ and 〈*O*〉 ≡ *f*(*Y*). Expanding *f* (*Y*) around the critical point *Y* → ∞, we thus get

7 $$\langle O\rangle ={f}_{0}+\frac{{f}_{1}}{Y}+\frac{{f}_{2}}{{Y}^{2}}+\cdots ,$$

where *f*_0_, *f*_1_, *f*_2_, … are expansion coefficients and should be constant. So the steady state average of *O* around the critical point is

8 $$\delta \langle O\rangle \equiv \langle O\rangle -{\langle O\rangle }_{c}\propto {f}_{1}{(\gamma -{\gamma }_{c})}^{(p-1)/p}+{f}_{2}{(\gamma -{\gamma }_{c})}^{2(p-1)/p}+\cdots .$$

Here 〈*O*〉_*c*_ is the steady state average of *O* at the exceptional point. Then the susceptibility of *O* is

9 $$\chi =\frac{\partial \langle O\rangle }{\partial \gamma }\propto {f}_{1}{(\gamma -{\gamma }_{c})}^{-1/p}+{f}_{2}{(\gamma -{\gamma }_{c})}^{-2/p+1}+\cdots .$$

For different observables, the expansion coefficients in Equation (7) are different. In particular, some of the expansion coefficients may vanish. We keep only the leading order singularity. Considering such a case, we thus have the following corollaries.

### Corollary 1

The steady state average of an arbitrary physical observable *O* at the non-Hermitian phase transition point presents scaling behavior 〈*O*〉 − 〈*O*〉_*c*_ ∝ _1_(*γ* − *γ*_*c*_)^*α*^ with exponent *α* being (1 − 1/*p*).

### Corollary 2

The susceptibility of an arbitrary physical observable in the steady state at the non-Hermitian phase transition point scales as *χ* − *χ*_*c*_ ∝ (*γ* − *γ*_*c*_)^*β*^ with exponent *β* being (−1/*p*).

For *p* = 2 case, Two levels coalesce at the exceptional point and we then have, 〈*O*〉 − 〈*O*〉_*c*_ ∝ _1_(*γ* − *γ*_*c*_)^*α*^ with exponent $\alpha =\frac{1}{2}$ and the susceptibility near the non-Hermitian phase transition point scales as, *χ* − *χ*_*c*_ ∝ (*γ* − *γ*_*c*_)^*β*^ with exponent $\beta =-\frac{1}{2}$. This means that the first derivative of an arbitrary physical quantity diverges at a behavior *χ* − *χ*_*c*_ ∝ (*γ* − *γ*_*c*_)^−1/2^ in non-Hermitian phase transition point. This reveals how the non-Hermitian coalescence in a finite system leads to the non-analytic behavior of physical observable, thus non-Hermitian phase transitions.

### Quantum Entanglement in Non-Hermitian Systems

Quantum entanglement provides a powerful way to understand the nature of many-body systems. In particular, it has been shown that entanglement is deeply related to phase transitions in condensed matter systems^42^. Recently it was also found that the entanglement in non-Hermitian phase transitions is bigger than that of Hermitian quantum phase transitions^33^. We first establish a theorem which connects the quantum entanglement and quantum phase transitions in non-Hermitian systems.

### Theorem 2

Any entanglement measure in the non-degenerate steady state of a non-Hermitian quantum many-body system with Hamiltonian *H*(*λ*) = *H*_0_ + *iγH*_1_ is a universal function of first derivative of steady state energy with respect to the control parameter, *M*(*λ*) = *M* $(\,\tfrac{\partial {E}_{S}}{\partial \gamma })$.

Relations between entanglement and quantum phase transitions in Hermitian models from density functional theory are established in ref. 43 and were generalized to finite temperatures by one of the authors^44^. Here we show that the density functional theory in non-Hermitian system uncovers deeper information about quantum entanglement and quantum phase transitions than that in Hermitian systems^43, 44^.

Since entanglement for a physical state can only be finite and near non-Hermitian phase transition point $Y=\frac{(\partial {E}_{S}(\gamma ))}{(\partial \gamma )}\propto {(\gamma -{\gamma }_{c})}^{(1-p)/p}$ diverges, then we can expand the entanglement measure around the non-Hermitian phase transition point by

10 $$M(Y)={m}_{0}+\frac{{m}_{1}}{Y}+\frac{{m}_{2}}{{Y}^{2}}+\cdots .$$

Here *m*_0_, *m*_1_, *m*_2_, … are the expansion coefficients and should be constant. So the entanglement around the non-Hermitian phase transition point scales with the control parameter as

11 $$\delta M=M(\gamma )-M({\gamma }_{c})\propto {m}_{1}{(\gamma -{\gamma }_{c})}^{(p-1)/p}+{m}_{2}{(\gamma -{\gamma }_{c})}^{2(p-1)/p}+\cdots ,$$

where *M*(*γ*_*c*_) is the steady state entanglement at the exceptional point. Then the first derivative of the entanglement measure scales as

12 $$\frac{\partial M}{\partial \gamma }\propto {m}_{1}{(\gamma -{\gamma }_{c})}^{-1/p}+{m}_{2}{(\gamma -{\gamma }_{c})}^{1-2/p}+\cdots .$$

The expansion coefficients in Equation (10) are different for different entanglement measures. In particular, some of the expansion coefficients may vanish. We retain the leading order singularity only. Considering such a case, we thus have.

### Corollary 3

Any entanglement measure of the steady state near the non-Hermitian phase transition point scales as *δM* = *M*(*γ*) − *M*(*γ*_*c*_) ∝_1_ (*γ* − *γ*_*c*_)^*μ*^ with exponent *μ* being (1 − 1/*p*).

### Corollary 4

The first derivative of any entanglement measure of the steady state near the non-Hermitian phase transition point scales as $\frac{\partial M}{\partial \gamma }-\frac{\partial M}{\partial \gamma }{|}_{\gamma ={\gamma }_{c}}\propto {(\gamma -{\gamma }_{c})}^{\nu }$ with exponent *ν* being (−1/*p*).

Theorem 2 and corollary 3 and 4 establish rigorously the connections between quantum entanglement and quantum phase transition in non-Hermitian systems. They are valid for any finite interacting spin systems and Fermions or Bosons in a lattices.

## Discussion

To illustrate the above idea, we study the LMG model with the Hamiltonian^33, 45^

13 $$H=\frac{V}{N}({J}_{x}^{2}-{J}_{y}^{2})-\frac{i{\rm{\Gamma }}}{2}{J}_{z}-\frac{i{\rm{\Gamma }}N}{2},$$

where *V* is the coupling strength and ${J}_{\alpha }\equiv \frac{1}{2}\sum _{i=1}^{N}{\sigma }_{i}^{\alpha },\alpha =x,y,z$ are the collective spin operators or spin polarization of all the atoms in the *α* = *x*, *y*, *z*, direction. LMG model is the simplest long-range interacting spin models. It is used to describe the magnetic properties of molecules such as Mn_12_ ancetate^46^. LMG model also captures the physics of interacting bosons in a double well structure^47, 48^ and is thus related to Bose-Einstein condensation and Josephson junction. We consider *V* as fixed and Γ as varying parameter. In terms of the raising and lowering operators of the collective spin, *J*_±_ = *J*_*x*_ ± *iJ*_*y*_, we have

14 $$H/V=\frac{1}{4N}({J}_{+}^{2}+{J}_{-}^{2})-\frac{i{\rm{\Gamma }}}{2}{J}_{z}-\frac{i{\rm{\Gamma }}N}{2}.$$

Here *γ* = Γ/*V* being dimensional control parameter. For convenience, we focus on the Dicke manifold with maximum angular momentum, so the Hilbert space has dimension *N* + 1. The Hermitian part of the Hamiltonian Eq. (14) can be experimentally realized through trapped ions^49, 50^ or cavity QED^51^. To realize the non-Hermitian part, we assume that the upstate has a finite lifetime with linewidth *γ*. In the absence of a decay event, the atoms evolution are governed by the Hamiltonian Eq. (14)^35–39^. In reality, one would perform the experiment many times and the case without decay event to realize Equation (14)^32, 33^.

**Figure 1:**
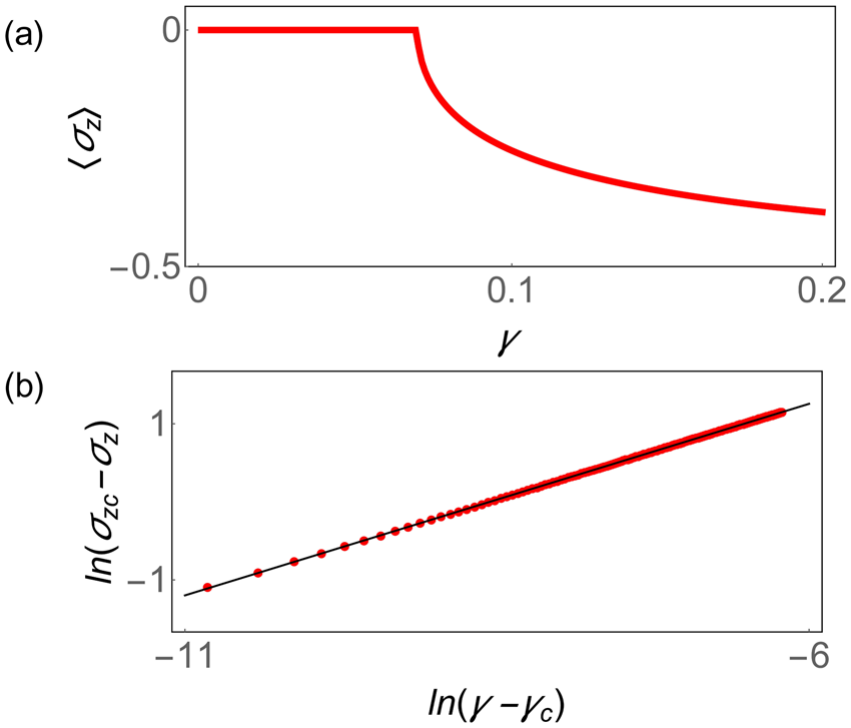
Quantum Phase transition in a Non-Hermitian LMG model. (**a**) The average magnetization along *z* axis,〈*σ*_*z*_〉 = 〈*J*_*z*_〉/*N*, as a function of *γ* in the LMG model with *N* = 40 spins. (**b**) Scaling of the magnetization around the critical point. The vertical axis plots $\mathrm{ln}\,({\langle {\sigma }_{z}\rangle }_{c}-\langle {\sigma }_{z}\rangle )$ with 〈*σ*_*z*_〉_*c*_ being the average of *σ*_*z*_ at the critical point. The horizontal axis is $\mathrm{ln}(\gamma -{\gamma }_{c})$, where *γ*_*c*_ is the critical control parameter. The red solid circle presents the numerical exact solution and the black solid line is the linear fitting line, where the slope is 0.49 ± 0.01.

Figure 1(a) shows the steady state average value of 〈*σ*_*z*_〉 = 〈*J*_*z*_〉/*N* in the LMG model with *N* = 40 spins as a function of the control parameter *γ*. One can see that there is a critical point *γ*_*c*_. If *γ* < *γ*_*c*_,〈*σ*_*z*_〉 = 0 and being smaller than zero if *γ* > *γ*_*c*_. In Fig. 1(b), we study the critical exponents of 〈*σ*_*z*_〉 and plot $\mathrm{ln}\,({\langle {\sigma }_{z}\rangle }_{c}-\langle {\sigma }_{z}\rangle )$ as a function of $\mathrm{ln}(\gamma -{\gamma }_{c})$ near the critical point. We made a linear fit and found that the critical exponents being 0.49 ± 0.01. And it indicates near the critical point *δ*〈*σ*_*z*_〉 ∝ (*γ* − *γ*_*c*_)^1/2^. This is consistent with the prediction from Corollary 1 since two levels coalesce at the critical point in the LMG model^33^.

To quantify many-body entanglement, we study the averaged quantum Fisher information which is defined by refs 52 and 53,

$$F=\frac{4}{3{N}^{2}}[{({\rm{\Delta }}{J}_{x})}^{2}+{({\rm{\Delta }}{J}_{y})}^{2}+{({\rm{\Delta }}{J}_{z})}^{2}],$$

where *N* is the number of spins. The multipartite entanglement that the quantum Fisher information detects has an immediate interpretation as a resource for quantum metrology^52, 53^.

**Figure 2:**
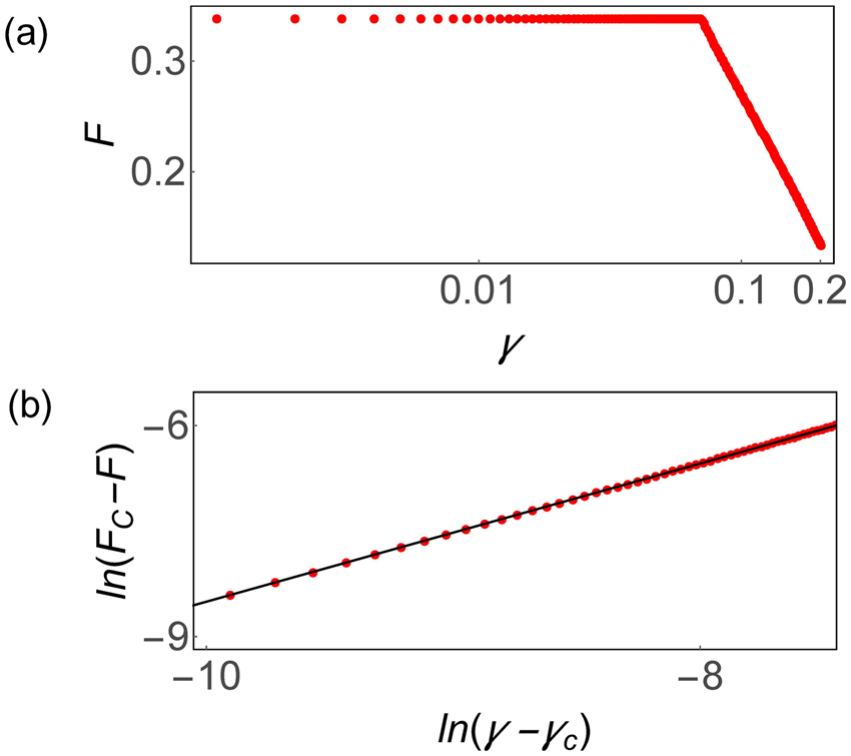
Multipartite entanglement in non-Hermitian phase transition. (**a**) Quantum Fisher information *F* as a function of the control parameter *γ* in the non-Hermitian LMG model for *N* = 40 spins. (**b**) Scaling of quantum Fisher information near the non-Hermitian phase transition point. The vertical axes is $\mathrm{ln}({F}_{C}-F)$ with *F*_*C*_ being the quantum Fisher information at the critical point and *F* the quantum Fisher information near the critical point and the horizontal axes is $\mathrm{ln}(\gamma -{\gamma }_{c})$. The red solid circle presents the numerical exact solution and the black solid line is the linear fitting line, where the slope is 0.98 ± 0.01.

In Fig. 2(a), we present the quantum Fisher information of the steady state in the non-Hermitian LMG model with *N* = 40 spins as a function of the control parameter. One can see that the quantum Fisher information is maximum when *γ* < *γ*_*c*_ and decreases when *γ* > *γ*_*c*_. In Fig. 2(b), we study how the quantum Fisher information scales near the critical point where the quantum Fisher information is maximum and denoted by *F*_*C*_. We plot $\mathrm{ln}({F}_{C}-F)$ as a function of $\mathrm{ln}(\gamma -{\gamma }_{c})$ near the critical point. We made a linear fit and found that the critical exponents being 0.98 ± 0.01. And it indicates near the critical point *F*_*C*_ − *F* ∝ (*γ* − *γ*_*c*_)^1^. This is consistent with the prediction from Corollary 3 since two levels coalesce at the critical point^33^.

### Summary

In this work we have uncovered universal critical behaviors for quantum phase transitions and quantum entanglement in non-Hermitian many-body systems from density functional theory perspective. We prove that the non-degenerate steady state of a non-Hermitian quantum many-body system is a universal function of the first derivative of the steady state energy with respect to the control parameter. This finding bridges the non-analytic behavior of physical observable with non-analytic behavior of steady state energy and explains why the quantum phase transitions in non-Hermitian systems occurs in finite systems and predicts universal scaling behavior of any physical observable and quantum entanglement near the non-Hermitian phase transition point. These results provide profound connections between entanglement and phase transition in non-Hermitian quantum many-body physics and may establish foundations for quantum metrology using non-Hermitian systems.

## Methods

Proof of Theorem 1 are based on the following two Lemmas:

### Lemma 1

There is a one-to-one correspondence between the non-degenerate eigenket $|{{\rm{\Psi }}}_{S}\rangle$ of the steady state in a non-Hermitian quantum many-body system with Hamiltonian *H*(*λ*) = *H*_0_ + *iγH*_1_ and the control parameter *γ*.

Proof. For a given *γ*, by diagonalizing *H*(*γ*) = *H*_0_ + *iγH*_1_, we can get the steady state $|{{\rm{\Psi }}}_{S}\rangle$. We also need to prove that the non-degenerate steady state also uniquely specifies the control parameter *γ*. This is done by reductio ad absurdum. We assume that two different parameters *γ* and *γ*′ with *γ* ≠ *γ*′ have the same steady state, $|{{\rm{\Psi }}}_{S}\rangle$, then we have two eigenvalue equations, $({H}_{0}+i\gamma {H}_{1})|{{\rm{\Psi }}}_{S}\rangle =E(\gamma )|{{\rm{\Psi }}}_{S}\rangle$ and $({H}_{0}+i\gamma ^{\prime} {H}_{1})|{{\rm{\Psi }}}_{S}\rangle =E(\gamma ^{\prime} )|{{\rm{\Psi }}}_{S}\rangle$. Subtracting these two equations, we get $i(\gamma -\gamma ^{\prime} ){H}_{1}|{{\rm{\Psi }}}_{S}\rangle =(E(\gamma )-E(\gamma ^{\prime} ))|{{\rm{\Psi }}}_{S}\rangle$. This means that $|{{\rm{\Psi }}}_{S}\rangle$ is also an eigenket of *H*_1_ or *γ* = *γ*′. But [*H*, *H*_1_] ≠ 0, $|{{\rm{\Psi }}}_{S}\rangle$ cannot be an eigenket of *H*_1_. We thus have *γ* = *γ*′. This contradicts the assumption. Therefore Lemma 1 is proved. Since $\langle {\tilde{{\rm{\Psi }}}}_{S}|$ is the eigenbra of the steady state of *H*(*γ*) with minimum imaginary part, likewise, we can prove that $\langle {\tilde{{\rm{\Psi }}}}_{S}|$ and *γ* are also one-to-one mapped.

### Lemma 2

There is a one-to-one map between the control parameter *γ* and the density ${\langle {H}_{1}\rangle }_{B}=\langle {\tilde{{\rm{\Psi }}}}_{S}(\gamma )|{H}_{1}|{{\rm{\Psi }}}_{S}(\gamma )\rangle$ in the non-degenerate steady state.

Proof. For a given *γ*, $\langle {\tilde{{\rm{\Psi }}}}_{S}(\gamma )|$ and $|{{\rm{\Psi }}}_{S}(\gamma )\rangle$ are uniquely specified according to Lemma 1. Then ${\langle {H}_{1}\rangle }_{B}=$ $\langle {\tilde{{\rm{\Psi }}}}_{S}(\gamma )|{H}_{1}|{{\rm{\Psi }}}_{S}(\gamma )\rangle$ can be determined. We denote the eigen kets of *H* at parameters *γ* and *γ*′ by $|{{\rm{\Psi }}}_{S}\rangle$ and $|{{\rm{\Psi }}^{\prime} }_{S}\rangle$, respectively and the eigen bras of *H* at parameters *γ* and *γ*′ by $\langle {\tilde{{\rm{\Psi }}}}_{S}|$ and $\langle {\tilde{{\rm{\Psi }}}}_{S}^{^{\prime} }|$, respectively. Now we have to show that if *γ* ≠ *γ*′, ${\langle {H}_{1}\rangle }_{B}\ne {\langle {H}_{1}\rangle }_{B}^{^{\prime} }$. This can be done by reductio ad absurdum. We assume two different control parameter *γ* ≠ *γ*′ produce the same density ${\langle {H}_{1}\rangle }_{B}={\langle {H}_{1}\rangle }_{B}^{^{\prime} }$, i.e. $\langle {\tilde{{\rm{\Psi }}}}_{S}|{H}_{1}|{{\rm{\Psi }}}_{S}\rangle =\langle {\tilde{{\rm{\Psi }}}}_{S}^{^{\prime} }|{H}_{1}|{{\rm{\Psi }}}_{S}^{^{\prime} }\rangle$. According to maximum of the imaginary part of the steady state energy, we have $\text{Im}\langle {\tilde{{\rm{\Psi }}}}_{S}|H(\gamma )|{{\rm{\Psi }}}_{S}\rangle  > \text{Im}\langle {\tilde{{\rm{\Psi }}}}_{S}^{^{\prime} }|H(\gamma )|{{\rm{\Psi }}}_{S}^{^{\prime} }\rangle$, which leads to an inequality, $\text{Im}{E}_{S} > \text{Im}{E}_{S}^{^{\prime} }+\text{Im}\,(i(\gamma -\gamma ^{\prime} )\langle {\tilde{{\rm{\Psi }}}}_{S}^{^{\prime} }|{H}_{1}|{{\rm{\Psi }}}_{S}^{^{\prime} }\rangle )$. Similarly by exchanging *γ* and *γ*′ and their eigenstates, we get another inequality, $\text{Im}{E}_{S}^{^{\prime} } > \text{Im}{E}_{S}+\text{Im}\,(i(\gamma ^{\prime} -\gamma )\langle {\tilde{{\rm{\Psi }}}}_{S}|{H}_{1}|{{\rm{\Psi }}}_{S}\rangle )$. Summing up two inequalities, we get a contradiction, $\text{Im}{E}_{S}^{^{\prime} }+\text{Im}{E}_{S} > \text{Im}{E}_{S}^{^{\prime} }+\text{Im}{E}_{S}$. Thus our assumption is wrong and Lemma 2 is proved.

### Proof of Theorem 1

Combining Lemma 1 and Lemma 2, we know that the non-degenerate steady state of a non-Hermitian quantum many-body system is uniquely specified by the density ${\langle {H}_{1}\rangle }_{B}=\langle {\tilde{{\rm{\Psi }}}}_{S}(\gamma )|{H}_{1}|{{\rm{\Psi }}}_{S}(\gamma )\rangle$. Hellmann-Feynman Theorem for non-Hermitian system tells us for any eigenstate of *H*(*γ*)^9^, $\langle {\tilde{{\rm{\Psi }}}}_{n}(\gamma )|\frac{\partial H}{\partial \gamma }|{{\rm{\Psi }}}_{n}(\gamma )\rangle =\frac{\partial {E}_{n}}{\partial \gamma }$. Applying Hellmann-Feynman Theorem for the steady state of non-Hermitian system, we get 〈*H*_1_〉_*B*_ = −*i* $\,\frac{\partial {E}_{S}}{\partial \gamma }$. Therefore the non-degenerate steady state, $|{{\rm{\Psi }}}_{S}\rangle$ is uniquely specified by $\,\frac{\partial {E}_{S}}{\partial \gamma }$. Theorem 1 is proved.

### Proof of Theorem 2

The proof follows from the fact that, according to Theorem 1, the steady state $|{{\rm{\Psi }}}_{S}\rangle$ in non-Hermitian systems is a unique function of $\,\frac{\partial {E}_{S}}{\partial \gamma }$ and also $|{{\rm{\Psi }}}_{S}\rangle$ provides the complete information of the system in the steady state, everything else is a unique function of $\,\frac{\partial {E}_{S}}{\partial \gamma }$. Formally let us consider an *n*-partite entanglement in spin-1/2 systems. For other cases, the proof can be generalized immediately. First of all any entanglement measure of *n*-qubits is always a function of the matrix elements of the reduced density matrix of these qubits, *M*(*ρ*_12_ …_*n*_). For spin-1/2 systems, the *n*-body reduced density matrix can be written as ${\rho }_{12\cdots n}={\sum }_{{a}_{1}{a}_{2}\cdots {a}_{n}=0,x,y,z}{C}_{{a}_{1}{a}_{2}\cdots {a}_{n}}{\sigma }_{1}^{{a}_{1}}{\sigma }_{2}^{{a}_{2}}\cdots {\sigma }_{n}^{{a}_{n}}$ and the expansion coefficients are given by ${C}_{{a}_{1}{a}_{2}\cdots {a}_{n}}={{\rm{Tr}}}_{12\cdots n}[{\rho }_{12\cdots n}{\sigma }_{1}^{{a}_{1}}{\sigma }_{2}^{{a}_{2}}\cdots {\sigma }_{n}^{{a}_{n}}]={\rm{Tr}}[{\rho }_{S}{\sigma }_{1}^{{a}_{1}}{\sigma }_{2}^{{a}_{2}}\cdots {\sigma }_{n}^{{a}_{n}}]=\langle {\sigma }_{1}^{{a}_{1}}{\sigma }_{2}^{{a}_{2}}\cdots {\sigma }_{n}^{{a}_{n}}\rangle$. Here *a*_1_, *a*_2_, … takes value of *0*, *x*, *y*, *z* with *σ*_0_ = *I* and ${\rho }_{S}=|{{\rm{\Psi }}}_{S}\rangle \langle {{\rm{\Psi }}}_{S}|$. According to Theorem 1, the average value of any observable can be taken as a function of $\,\frac{\partial {E}_{S}}{\partial \gamma }$. Therefore, any entanglement measure is a function of $\,\frac{\partial {E}_{S}}{\partial \gamma }$ and Theorem 2 is proved.
